# Supplementary material for: Upregulation of TRPM3 in nociceptors innervating inflamed tissue
Source: eLife. 2020 Sep 3;9:e61103. doi: 10.7554/eLife.61103 (PMC7470828; doi:10.7554/eLife.61103)
Supplement: Transparent reporting form [file elife-61103-transrepform.pdf]

eLIFE
1st Floor
24 Hills Road
Cambridge CB2 1JP, UK
P 01223 855340
W elifesciences.org
T @elife

eLife's transparent reporting form

We encourage authors to provide detailed information *within their submission* to facilitate the interpretation and replication of experiments. Authors can upload supporting documentation to indicate the use of appropriate reporting guidelines for health-related research (see EQUATOR Network), life science research (see the BioSharing Information Resource), or the ARRIVE guidelines for reporting work involving animal research. Where applicable, authors should refer to any relevant reporting standards documents in this form.

If you have any questions, please consult our Journal Policies and/or contact us: editorial@elifesciences.org.

Sample-size estimation

- You should state whether an appropriate sample size was computed when the study was being designed
- You should state the statistical method of sample size computation and any required assumptions
- If no explicit power analysis was used, you should describe how you decided what sample (replicate) size (number) to use

Please outline where this information can be found within the submission (e.g., sections or figure legends), or explain why this information doesn't apply to your submission:

The following information is provided in the Methods Section, Statistical analysis: "No statistical methods were used to predetermine the number of animals used in this study; since no a priory data were available for the novel imaging approaches used in this work, a power calculation was not feasible. However, our sample sizes are similar to those generally employed in other studies in the field, and potential limitations due to insufficient power are discussed in the text."

Replicates

- You should report how often each experiment was performed
- You should include a definition of biological versus technical replication
- The data obtained should be provided and sufficient information should be provided to indicate the number of independent biological and/or technical replicates
- If you encountered any outliers, you should describe how these were handled
- Criteria for exclusion/inclusion of data should be clearly stated
- High-throughput sequence data should be uploaded before submission, with a private link for reviewers provided (these are available from both GEO and ArrayExpress)

Please outline where this information can be found within the submission (e.g., sections or figure legends), or explain why this information doesn't apply to your submission:

- Details on the number of individual experiments (individually analyzed mice) are provided in the legends of Figures 1,2, Figure 2 – figure supplement 1 and Figure 4. Data from individual skin assays are provided in Figure 4 – figure supplement 1.
- The precise number of analyzed neurons is provided in the legends of Figures 1 and 2, Figure 2 – figure supplement 1 and values for individual neurons are plotted in Figure 1 – figure supplement 2, Figure 2 – figure supplement 2.
- In the DRG calcium measurements, cells that did not respond to high K+ stimulation were excluded from the analysis. This is indicated in the legend of Figure 2.

Statistical reporting

- Statistical analysis methods should be described and justified
- Raw data should be presented in figures whenever informative to do so (typically when N per group is less than 10)
- For each experiment, you should identify the statistical tests used, exact values of N, definitions of center, methods of multiple test correction, and dispersion and precision measures (e.g., mean, median, SD, SEM, confidence intervals; and, for the major substantive results, a measure of effect size (e.g., Pearson's r, Cohen's d)
- Report exact p-values wherever possible alongside the summary statistics and 95% confidence intervals. These should be reported for all key questions and not only when the p-value is less than 0.05.

Please outline where this information can be found within the submission (e.g., sections or figure legends), or explain why this information doesn't apply to your submission:

- Statistical tests are described in the legends of Figures 1,2, Figure 2 – figure supplement 3, Figure 4 and Figure 4 – figure supplement 1.
- The choice of parametric and non-parametric tests is justified in the Methods Section, Statistical analysis: "Shapiro–Wilk test was used to test the normality of the data, determining whether parametric or non-parametric tests were applied. The specific parametric and non-parametric tests that were used are specified in the text and legends.."
- As outlined in the Methods Section, Statistical analysis: "Summary data for parametric datasets are shown as mean + SEM. We used bootstrapping with 10,000 bootstrap resamples to calculate 95% confidence intervals for non-parametric datasets."
- Exact p-values of all relevant comparisons are indicated in the figures.

(For large datasets, or papers with a very large number of statistical tests, you may upload a single table file with tests, Ns, etc., with reference to sections in the manuscript.)

Group allocation

- Indicate how samples were allocated into experimental groups (in the case of clinical studies, please specify allocation to treatment method); if randomization was used, please also state if restricted randomization was applied

- Indicate if masking was used during group allocation, data collection and/or data analysis

Please outline where this information can be found within the submission (e.g., sections or figure legends), or explain why this information doesn't apply to your submission:

- As outlined in the description of the experiments in the results section, all experiments were performed in mice where one hind paw was inflamed by injection of CFA, whereas the other paw served as vehicle control. Therefore, no specific allocation to experimental groups was required.
- As outlined in the Methods Section, Statistical analysis: "Imaging of the DRG and skin preparations and subsequent data analysis were performed by researchers that were blinded for the treatment (vehicle versus CFA)."

Additional data files ("source data")

- We encourage you to upload relevant additional data files, such as numerical data that are represented as a graph in a figure, or as a summary table
- Where provided, these should be in the most useful format, and they can be uploaded as "Source data" files linked to a main figure or table
- Include model definition files including the full list of parameters used
- Include code used for data analysis (e.g., R, MatLab)
- Avoid stating that data files are "available upon request"

Please indicate the figures or tables for which source data files have been provided:

Not applicable.

eLife Sciences Publications, Ltd is a limited liability non-profit non-stock corporation incorporated in the State of Delaware, USA, with company number 5030732, and is registered in the UK with company number FC030576 and branch number BR015634 at the address 1st Floor, 24 Hills Road, Cambridge CB2 1JP | August 2014
